# Condition Monitoring of Horizontal Sieving Screens—A Case Study of Inertial Vibrator Bearing Failure in Calcium Carbonate Production Plant

**DOI:** 10.3390/ma16041533

**Published:** 2023-02-12

**Authors:** Jacek Wodecki, Pavlo Krot, Adam Wróblewski, Krzysztof Chudy, Radosław Zimroz

**Affiliations:** Faculty of Geoengineering, Mining and Geology, Wroclaw University of Science and Technology, Na Grobli 15, 50-421 Wroclaw, Poland

**Keywords:** sieving screen, raw materials processing, vibrations, condition monitoring, bearings diagnostics, failure

## Abstract

Predictive maintenance is increasingly popular in many branches, as well as in the mining industry; however, there is a lack of spectacular examples of its practice efficiency. Close collaboration between Omya Group and Wroclaw University of Science and Technology allowed investigation of the failure of the inertial vibrator’s bearing. The signals of vibration are captured from the sieving screen just before bearing failure and right after repair, when it was visually inspected after replacement. The additional complication was introduced by the loss of stable attachment of the vibrator’s shield, which produced great periodical excitation in each place of measurement on the machine. Such anomalies in the signals, in addition to falling pieces of material, made impossible the diagnostics by standard methods. However, the implementation of advanced signal processing techniques such as time–frequency diagrams, envelope spectrum, cyclic spectral coherence, orbits analysis, and phase space plots allowed to undermine defects (pitting on the inner ring). After repair, the amplitudes of vibration from the damaged bearing side were reduced by five times, while sound pressure was only two times lower. The quantitative parameters of vibrations showed significant changes: time series RMS (−68%) median energy of spectrograms (89%), frequencies ratio of cyclic spectral coherence (−85%), and average amplitude of harmonics in envelope spectrum (−80%). The orbits demonstrated changes in inclination angle (16%) and sizes (−48, … −96%), as well as phase space plots sizes (−28, … −67%). Directions of further research are considered.

## 1. Introduction

Predictive maintenance based on advanced measurement tools and methods has become more and more popular in many branches, as well as in the mining industry; however, there is a lack of spectacular examples of its practice efficiency. Most examples of diagnostic methods development and validation are related to laboratory test rigs, while the rare cases are analyzed in different unique machines such as bucket wheel excavators [1], wind turbines [2], compressors [3], conveyors [4], and crushers [5] working under variable speed and load conditions, as well as excessive external disturbances.

Close collaboration between Omya Group and Wroclaw University of Science and Technology allowed the representation of unique material showing results of the analysis of vibration data captured from the industrial vibrating sieving screen just before and right after its repair. To evaluate screen technical conditions, up-to-date signal processing techniques are applied such as time–frequency methods, envelope spectrum, cyclic spectral coherence, and orbits analysis, including phase space plots. It was found that one of the bearings is damaged; moreover, the loss of stable attachment of the vibrators shield caused great excitation in the machine, which was a significant anomaly in the signals negatively influencing the results of diagnostics.

Condition monitoring of bearings in the sieving screen is a specific problem. To assure material classification (shaking of the screen), a serious unbalance is introduced to the shafts of vibrators driven by electric motors. So, signal by definition is time–varying and nonstationary. Moreover, the material stream feeding to the sieving screen consists of various size particles from sand-like to oversized (tens of centimeters) pieces of rock [6,7,8]. It generates massive impulsive noise with strongly non-Gaussian properties.

In addition, the special series of roller bearings used in vibrating screens and other types of heavy-duty industrial machines are always subjected to excessive shock impacts in case of radial clearances (backlashes) appearing. In this case, the surface layer of both inner and outer rings exhibits gradual alterations of microstructures that initiate microcracks and subsequent pitting. Detailed multiscale modeling of rolling cyclic fatigue in bearing elements is considered in [9]. Depending on the bearing design and its service time, hardening can take place at a depth of nearly 450 µm, while the softened layer is observed at 500–700 µm depth. The enlarged clearances, in their turn, cause shaft beating, deterioration of peripheral labyrinth sealing, and leak of lubrication. The machine maintenance staff of the investigated screen reported exactly all of the above-mentioned symptoms: lubrication oil leakage, wear of shaft neck, and excessive radial clearances, which were previously measured by calibrated gauges (0.5–0.6 mm between the shaft and the inner ring, about 0.8–1.0 mm between the outer ring and housing).

The permanent monitoring of bearing temperature is not provided in this vibrating screen, yet, instead, scheduled inspections with a manual pyrometer are conducted. The slightly increased temperature on the lower bearing from the drive side was noted. This bearing reached a temperature of approximately 11 °C higher than the upper shaft bearing. In the following 5 days between inspections, the temperature difference decreased to about 5 °C. On the opposite side of the screen, the temperature difference was 4 °C, and it increased to about 8 °C in the following 5 days. In addition, the recent problem has appeared in the form of bearing housing damage (unbalanced mass touching), which complicated the measurement of vibration due to periodic strong impacts near the bearings on the drive side.

Based on these observations, maintenance staff considered using more advanced analysis to support decision making about shaft repair and bearing replacement. This paper represents the results of the damaged bearing’s diagnostics by the vibration and partial sound signal.

The paper is organized as follows. First, we recall the most frequent challenges in sieving screen operation and the most popular techniques used to deal with them. Next, we describe the experimental work and machine. We present the results of measurements before and after replacement and the results of data analysis using the mentioned techniques. The last parts of the paper contain a visual inspection of the replaced bearing with race wear and discussion, as well as the final conclusion.

## 2. State of the Art

Predictive maintenance is rapidly growing, as well as in the raw material sector, including mining machines for excavating, drilling, transporting, processing (sieving and crushing), ventilation, and related technologies [10,11,12,13,14,15,16,17,18,19]. For example, the developments in vibration-based diagnostics of gear and bearings used in belt conveyors are given in a review [20]. The dynamics of tumbling mills and the energy efficiency analysis of copper ore ball mill drive systems are represented in [21]. Modern systems of condition monitoring can include such useful features as calculation of the remaining useful life of machine elements [22,23,24,25] combined with process optimization and control [26,27,28].

The problems of impulsive noise cancellation for copper ore crusher vibration signal enhancement are considered in [29]. Results of the development and industrial verification of the diagnostic model for the vibrating sieving screen are represented in [30].

For the diagnostics of bearings, the author in [31] proposed an infogram as a method to extract repetitive transients in the signals. Additional enhancement of this approach for non-Gaussian data is conducted in [32]. In [33], a new concept is proposed, called IESFOgram (Improved Envelope Spectrum via Feature Optimization), which is a gram for rolling element bearing diagnostics under nonstationary operating conditions. A comparison of advanced bearing diagnostic techniques may be found in [34].

An analysis of the kurtogram data presentation method’s performance is given in [35] in case of high-level non-Gaussian noise. An impulsive source separation technique based on a combination of Non-negative Matrix Factorization of bifrequency map, spatial denoising, and Monte Carlo simulation is proposed in [3]. The Second-Order Cyclostationary (CS2) analysis in presence of non-Gaussian background noise–effect on traditional estimators and resilience of log-envelope indicators is considered in [36]. Several advanced techniques, such as spectral kurtosis, spectral L2/L1 norm, spectral smoothness index, and spectral Gini index, for characterizing repetitive transients have been proposed by the author in [37].

An important theoretical and practical problem is informative frequency band selection. This is because the falling pieces of sieved material generate heavy-tailed impulsive non-Gaussian noise. A novel approach for this problem’s solution, based on the conditional variance statistic with application to bearing fault diagnosis, is proposed in [38,39]. The local damage detection method based on vibration data analysis in the presence of Gaussian and heavy-tailed impulsive noise is developed in [40]. A relatively simple but reliable method for condition monitoring of gearboxes operating in impulsive environments is proposed in [41]. This method uses synchronous averaging for non-Gaussian noise removal, however, the mean operator has been replaced by a median. It was also discovered in [42] that the source of non-Gaussian behavior may be related to strong electromagnetic interference. Bearing diagnostics under such conditions are resolved based on integrated spectral coherence. The model for simulation of the impulsive signals in the sieving screen is proposed in [43]. The procedures of identification, decomposition, and segmentation of impulsive vibration signals with deterministic components for the case of sieving screen are represented in [44].

The Teager–Kaiser energy operator (TKEO) and Hilbert Transform (HT) are widely used to demodulate signals (envelope spectrum analysis). However, these methods are sensitive to noise, hence have certain limitations in the vibrating screens. To address the problems of bearings diagnostics in vibrating screens, an alternative energy operator method named the envelope-derivative operator (EDO) is proposed in [45]. The authors in [46] constructed a multimodal feature matrix composed of different types of entropy. The early fault diagnosis of the screen exciter bearing is realized by using the support vector machine (SVM) improved by the Aquila optimizer algorithm (AO-SVM). Variational mode decomposition (VMD) and K-L divergence are applied in [47] to the bearing fault diagnosis of vibrating screens.

Since the vibrating sieving screens work due to cyclic excitation from the rotating unbalanced masses, they are categorized as cyclostationary systems. Hence, the modeling of such signals plays an important role in diagnostics [48]. Regarding the design of exciters, the main efforts are concentrated on obtaining the wider spectrum of vibration [49,50] and dynamic parameters tuning concerning sieved material properties (fraction size and humidity) [51]. However, the majority of heavy industrial horizontal sieving screens have almost a similar design with inertial vibrators, and new developments are reported to be only at the stage of laboratory testing, e.g., electromagnetic or hydraulic excitation actuators allowing easier process regulation; magnetorheological supports for the stiffness control; and multimotor inertial exciters.

The relationship between spectral correlation and envelope analysis in the diagnostics of bearing faults and other cyclostationary machine signals is described in [52]. Periodically impulsive behavior detection in noisy observation based on a generalized fractional-order dependency map is developed in [53]. A generalized spectral coherence is proposed in [54,55] for the detection of cyclostationarity in the signals with the alpha-stable distribution. Short-term spectral analysis and different modifications of discrete Fourier transform [56] is widely used as the most popular time–frequency representation [57]. A detailed tutorial on rolling bearings diagnostics can be found in [58].

In the vibrating screening machines, the main parameter determining their performance, energy consumption, and output product quality is the trajectory (orbit) of sieving decks and bulk material particles’ movement. Additionally, orbits can also react to certain failures in bearings or supporting springs [59]. This parameter is used in the diagnostics of many rotating machines [60,61,62,63,64]. Reconstructing the shaft orbit using Instantaneous Angular Speed (IAS) measurement to detect bearing faults is proposed in [65]. Rotating machinery diagnostics using deep learning on orbit plot images is proposed in [66]. Experimental observations in the shaft orbits with different faults related to the rotor are represented in [67,68].

Due to using two orthogonal axes for screen motion measurement and following the integration of vibration accelerometers’ signals, the Phase Space Plots (PSP) can be built as a whole portrait of the dynamic system. In particular, PSP is useful for analysis of the bifurcations in nonlinear systems when they are susceptible to minor changes in parameters associated with fault development [69,70]. The application of the PSP technique needs the development of qualitative measures of trajectories topology [71]. In this paper, PSP is used to assess the faults in the bearings of vibrators.

According to our previous experience and investigation of industrial plants, the most frequently occurring failures in the vibrating screens are as follows:

- Vibrators’ bearings (heat and fatigue);
- Supporting springs (stiffness and cracks);
- Bolted joints (cracks and cyclic fatigue);
- Screening decks (abrasive wear);
- Inertial vibrators (rotation synchronization);
- Unbalanced masses (angular mismatch);
- Strengthening beams (cyclic fatigue);
- Electric drives (uneven load, starting resonances, and belts).

Currently, there are several condition monitoring systems in the market where options for the diagnostics of vibrating machines are declared.

- CONIQ (Schenck) is based on six-dimensional vibration data measured with piezoelectric accelerometers and bearings temperature.
- FAG SmartCheck (Schaeffler) recognizes such damages in a filled screen as loosening and breakage of springs; the monitored parameters are vibrations, temperature, and a load of drives.
- ScreenWatch (Check) (Metso) uses wireless self-powered vibration sensors and detects a deviation in screen motion caused by damaged springs and bearings, as well as the settings of unbalanced masses.
- Copperhead (SKF) detects the faults of gears, bearings, screen structure, and sieving decks.

All these systems are based on the standard algorithms of vibration monitoring aimed at the detection of local defects in the bearings. However, the falling copper ore is a source of random impulsive noise in vibration signals. The recently completed joint research project OPMO (Operation monitoring of mineral crushing machinery) funded by EIT RawMaterials resulted in an innovative condition monitoring system specialized for vibrating screen application, where the above-mentioned methods of nonstationary signals processing have been implemented for the diagnostics of bearings and supporting springs.

## 3. Methodology

**Figure 1:**
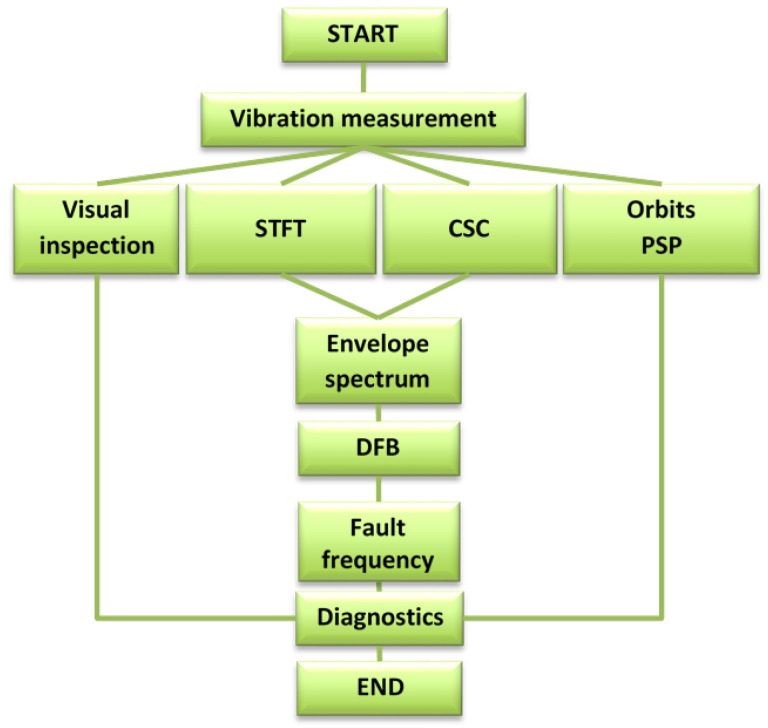
The scheme of diagnostics methodology: STFT—short FFT; CSC—cyclic spectral coherence; PSP—phase space plot; DFB—diagnostic frequency band.

The scheme of the research methodology is depicted in Figure 1. It includes vibration measurement, with accelerometers on the bearings of the vibrating screen taking signals in two orthogonal directions. Then, several methods are applied to the raw data, namely visual analysis of time series, short FFT, cyclo-maps, and orbits with phase space plots building. The envelope function is also appropriate for data analysis. Based on the results obtained in the frequency domain, the diagnostics frequency band is determined to contain different ranges, including the characteristic frequencies of bearings defects. After that, the conclusion about the sources of high-amplitude components is derived and the final diagnosis is formulated. Until the dismounting and inspection of machine units, the diagnosis remains to be a hypothesis.

### 3.1. Spectrogram

Short-Time Fourier Transform (STFT), which for one-dimensional discrete data $s_{m}=\left(s_{1}^{\left(m\right)},\cdots ,s_{N}^{\left(m\right)}\right)$, with m∈1,⋯,M is given by the formula [56]:

(1) $$\mathrm{STFT}\left(i,k\right)=\sum_{f=0}^{L-1}s\left[k+f\right]w\left[f\right]e^{-j2\pi if/I},$$

where *j* is the imaginary operator, 0≤i≤I−1 is the frequency bin for the *I* total frequency bins, k=0,⋯,K−1 is the time point for the *K* total time points, and w[.] is the window of length *L*. One can observe that in STFT for each time point the Fourier Transform is calculated using Fast Fourier Transform (FFT). Furthermore, the spectrogram is an absolute value of the STFT:

(2) Y(i,k)=Spec(i,k)=|STFT(i,k)|.

### 3.2. Envelope Spectrum Analysis

The procedure of diagnostic signal amplitude demodulation supposes that the modulating signal is carrying information about the damage. This is one of the simplest and most popular signal processing techniques for the detection of local faults. The idea of demodulation consists of appropriate frequency band selection, performing band-pass filtering for this band, and then using the Hilbert Transform to determine the envelope signal, in which the spectrum is then analyzed.

The Hilbert Transform (HT) of the real signal *x*(*t*) can be computed using such formula:

(3) $$h\left(t\right)=H\left\{x\left(t\right)\right\}=\frac{1}{\pi }\int_{-\infty }^{\infty }\frac{x(\tau )}{t-\tau }d\tau .$$

The magnitude of the analytical signal from the Hilbert Transform is as follows:

(4) $$x_{ht}\left(t\right)=\left|x(t)+j\cdot h(t)\right|=\sqrt{x^{2}\left(t\right)+h^{2}\left(t\right)}.$$

A Fourier Transform is then applied to this analytical signal:

(5) $$E\left(t\right)=\left|\mathrm{FT}(x_{ht}\left(t\right))\right|.$$

### 3.3. Cyclic Spectral Coherence

The Cyclic Spectral Coherence (CSC) is the bifrequency representation. It depends on the carrier frequency (*f*) and the modulation frequency (α). It was introduced by Antoni in 2007 [72]. The Cyclic Power Spectrum (CPS) $S_{X}\left(f;\alpha \right)$ of the signal x can be described by the following formula:

(6) $$S_{X}\left(f;\alpha \right)=\lim_{L\to \infty }\frac{1}{L}E\left(F_{x,L}\left(f+\frac{\alpha }{2}\right)\bar{F_{x,L}\left(f-\frac{\alpha }{2}\right)}\right),$$

where $F_{x,L}\left(f\right)$ is the Fourier transform of the signal x calculated on the interval of length *L*. In the cyclostationary signal, for some of the modulation frequency α≠0, the CPS is expected to meet the condition $\left|S_{X}\left(f;\alpha \right)\right|>0$. Based on the Equation (6), the formula of CSC can be introduced [72]:

(7) $$\mathrm{CSC}\left(f;\alpha \right)={\left|\gamma_{X}\left(f;\alpha \right)\right|}^{2}=\frac{{\left|S_{X}\left(f;\alpha \right)\right|}^{2}}{S_{X}\left(f+\frac{\alpha }{2};0\right)S_{X}\left(f-\frac{\alpha }{2};0\right)}.$$

The CSC is a useful tool to analyze the cyclostationarity of the signal. It allows for determining the strength of the cyclic spectral autocorrelation of the signal. The CSC statistic is normalized and its values range between 0 and 1. When the ${\left|\gamma_{X}\left(f;\alpha \right)\right|}^{2}$ is significantly higher than 0, then the signal reveals the cyclostationarity property at carrier frequency *f*, with a modulation period equal to T=1/α.

Following the Equation (7), the CSC estimation is performed with an estimator of CPS. The estimator of SC is given by the formula:

(8) $${\left|{\hat{\gamma }}_{X}\left(f;\alpha \right)\right|}^{2}=\frac{{\left|{\hat{S}}_{X}\left(f;\alpha \right)\right|}^{2}}{{\hat{S}}_{X}\left(f+\frac{\alpha }{2};0\right){\hat{S}}_{X}\left(f-\frac{\alpha }{2};0\right)},$$

where ${\hat{S}}_{X}\left(f;\alpha \right)$ is an estimator of the CPS [72].

### 3.4. Orbits and Phase Space Plots

Orbit analysis is a necessary tool for the analysis of vibrating rotating machinery. The process is essentially an extension of time waveform analysis plotting time-domain data from a pair of orthogonal probes on an orbit graph with consideration for the physical location of the sensors (accelerometers or proximity probes).

In our case of acceleration measurements by the orthogonal vibration sensors, the original signals are sequentially integrated to obtain velocity and displacement signals. Since the integration accumulates the bias in the signals, this operation needs its removal by linear or nonlinear detrending operations. The final graph is constructed in the coordinates of Horizontal Displacement (abscissa X) and Vertical Displacement (ordinate Y).

In addition to orbits analysis, the Phase Space Plots (PSP) are also considered in this paper as a possible tool for measurement data analysis in diagnostic procedures. In distinction to orbits, the final graph is constructed in the coordinates of Displacement (abscissa X) and Velocity (ordinate Y).

## 4. Experiments

**Figure 2:**
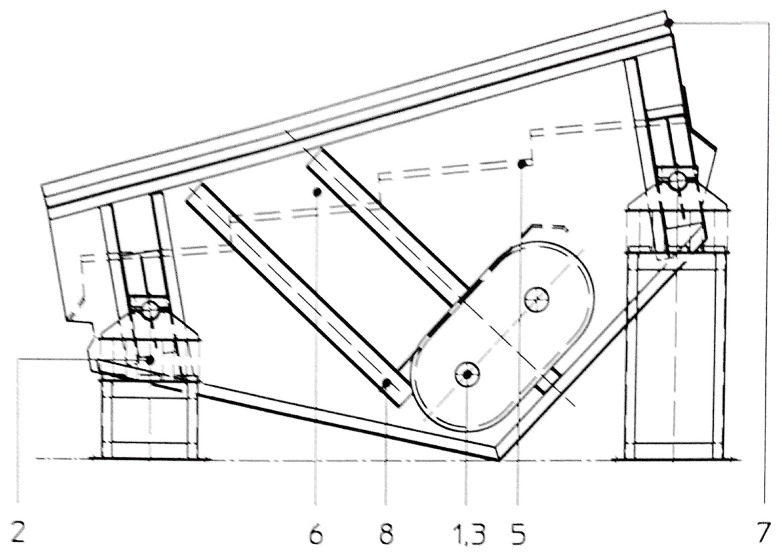
The overall side view of the vibrating sieving screen.

**Figure 3:**
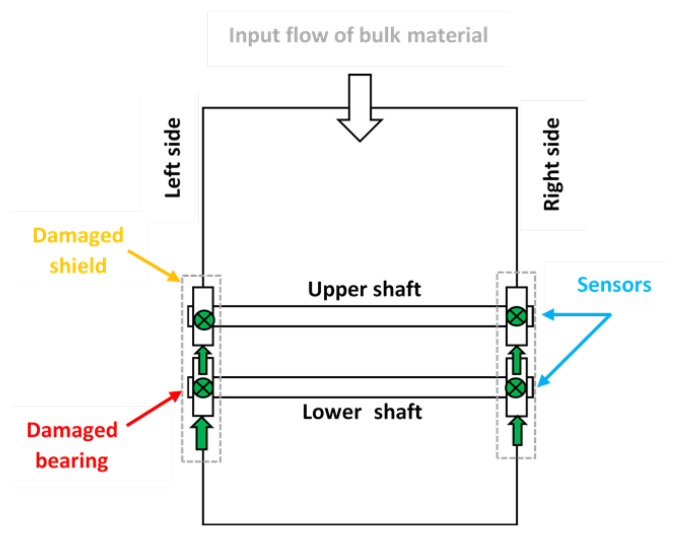
The overall top view with sensors placement and position of damaged bearing and vibrator shield.

The whole view of the investigated vibrating sieving screen is shown in Figure 2. It consists of shafts with bearings (1, 3); Cardan couplings (4); spring supports (2); grizzly deck (5); side walls with protection (6); and reinforced bars (7). Electric motors of 30 kW power and 1470 RPM rotation speed (situated on the opposite side) are connected with two unbalanced vibrators by the Cardan shafts, providing technological excitation of the screen. The vibrators are dynamically synchronized without kinematic links between them. This is the most typical design of vibrating screens used in the calcium carbonate plant of Omya Group and other enterprises of the mineral processing industry. The only difference there can be is belt drives instead of Cardan shafts. The overall top view of the screen with sensors placement is shown in Figure 3.

**Figure 4:**
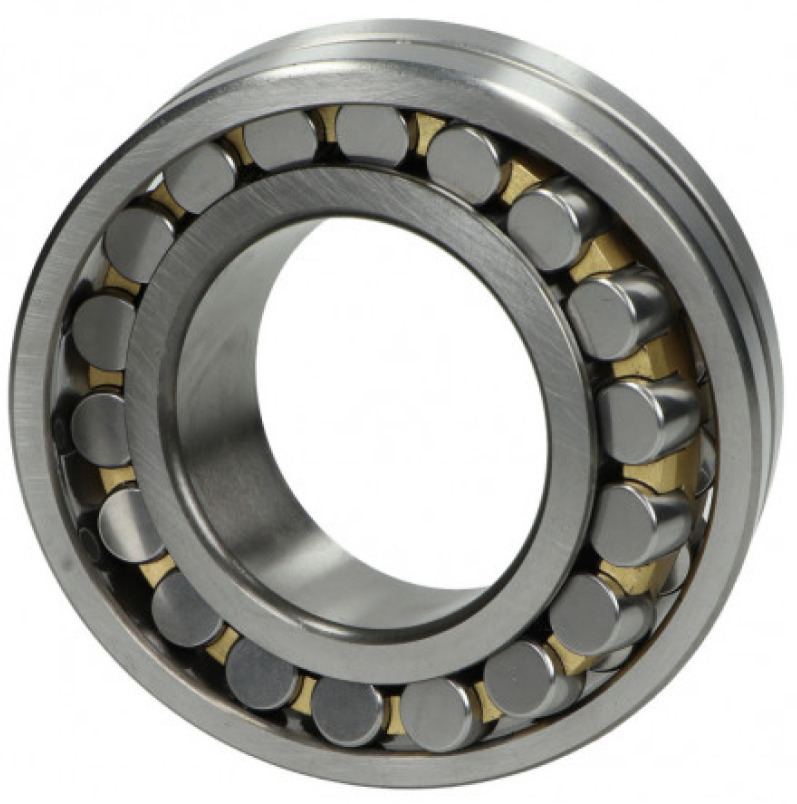
The special series spherical roller bearings (SKF22324VAJ): basic static load—1120 kN; dynamic load—850 kN; and limiting speed—2000 RPM.

The special series SKF 22234 VAJ spherical roller bearings (see Figure 4) installed on the vibrators’ shafts can accommodate heavy loads in both directions. They are self-aligning and compensate shaft misalignment and deflections, with virtually no increase in friction or temperature. Nevertheless, these elements of vibrators are subjected to high loads and cyclic fatigue. By the specification of the producer (SKF), bearings for the vibrating sieving screen have greater than normal clearances, which play the most important role in their durability [73,74].

The experiments were conducted twice in one of the plants in the Omya Group. The first measurement trial was carried out just before the planned replacement of rolling element bearings, and the second one was right after the new bearings were installed.

**Figure 5:**
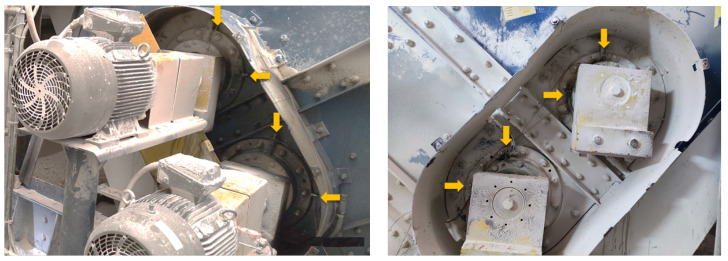
Vibration sensors’ (accelerometers) placement on the upper and lower shafts of the vibrating screen.

**Figure 6:**
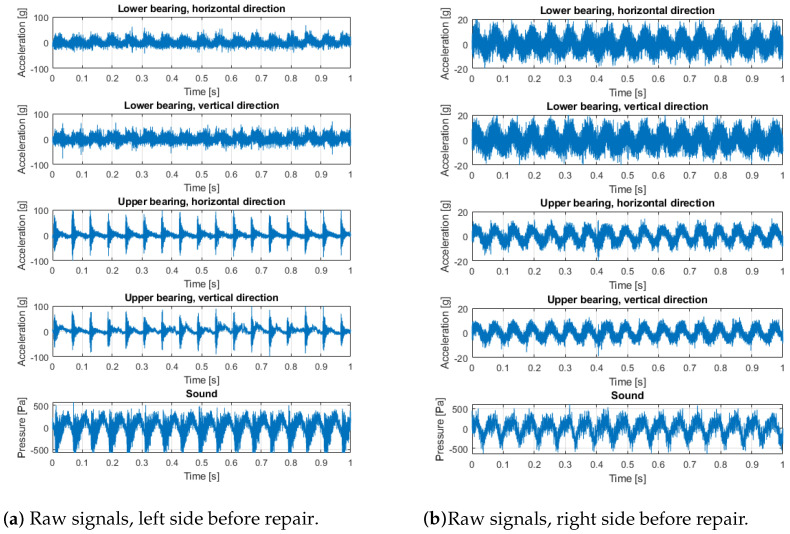
Raw signals: left and right side before repair.

**Figure 7:**
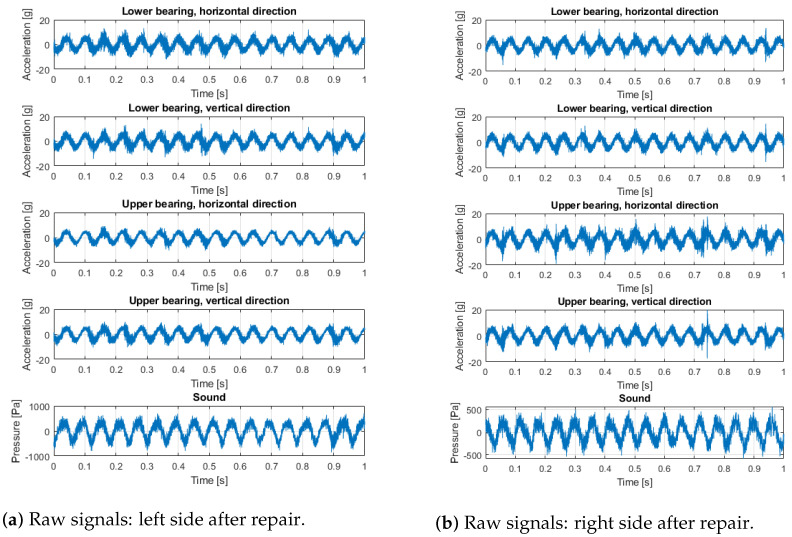
Raw signals: left and right side after repair.

Measurements were made using the Kistler LabAmp 5165A data acquisition system, Kistler 8702B500 accelerometers, and Bruel & Kjaer 4189 microphone. The places and directions of the accelerometers’ installation on both sides of the screen are shown in Figure 5. The sampling frequency of both vibration and sound signals was 50 kHz. The acoustic signal contains all effects from the loudest environmental sources. For this reason, the audio record is only used to validate the main machine operation cycles of shaft rotation observed in the vibration data, since the rotation sensors were not used in these experiments. The raw signals collected on the machine (before bearings replacement—with expected bearings problems—and after bearings replacement with new bearings installed) are presented in Figure 6 and Figure 7. The measurements were taken on the left and right sides of the screen, in two directions.

## 5. Data Analysis

**Figure 8:**
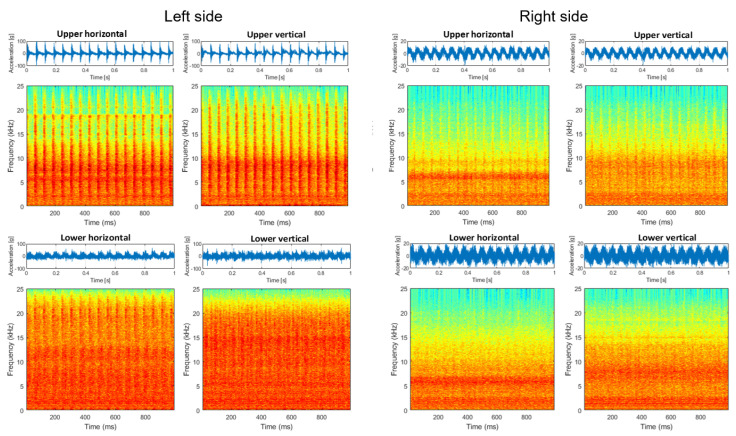
Signals and spectrograms before repair.

Signals from the screen are highly nonstationary, thus time–frequency analysis is a reasonable approach to understanding the properties of the signal. In Figure 8, signals and the corresponding spectrograms of these signals are presented. It is seen that signals from the left side (both horizontal and vertical directions of the upper left bearing) are highly dominated by cyclic impulses. The period of these impulses is related to rotational frequency, and the reasons for these impulses are mechanical shocks. It is also seen on the upper bearing on the right side (vibration transmission effect), as well as on the lower left bearing. This phenomenon is barely observed on the lower right bearing, as the distance between excitation and the sensor is the biggest in all cases. It is worth noticing that the lower left bearing generates a lot of noise (in the sense of amplitude as well as spectral content—it is more wide-band than other signals).

**Figure 9:**
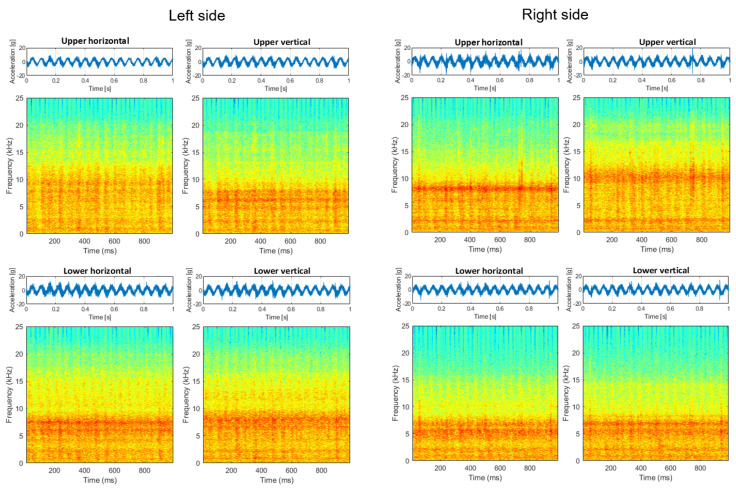
Signals and spectrograms after repair.

To compare signals before (Figure 8) and after repair with damaged bearing replacement (Figure 9), in the latter case, all time series are similar; there are some minor differences in time–frequency maps, but one may approximately conclude they are almost the same (level and spectral structure).

**Figure 10:**
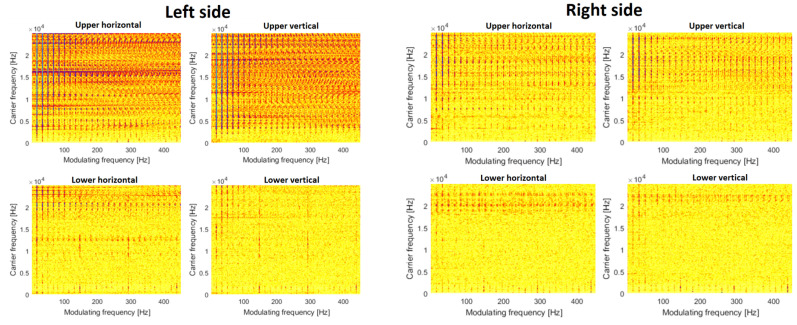
Cyclic spectral coherence (CSC) before repair.

**Figure 11:**
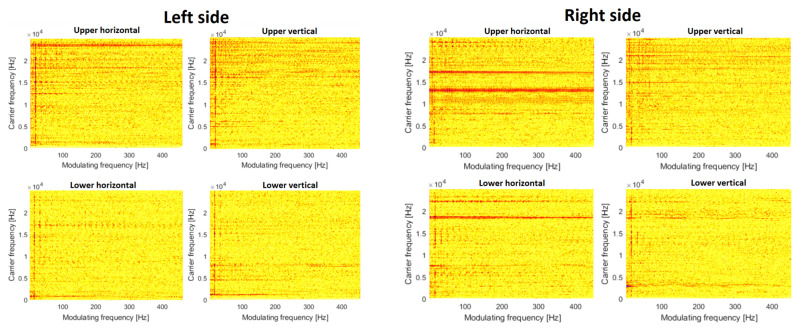
Cyclic spectral coherence (CSC) after repair.

The time–frequency map allows us to understand spectral content and its variation over time, but to identify nonlinear modulation between components, it is better to use the CSC map presented in Figure 10 and Figure 11. In Figure 10, by analogy to spectrograms, one may see a family of modulating harmonics, however, there are two sources of modulation. First, it is seen that cyclic impulses in the time domain cause amplitude modulation. The carrier band is really wide, and modulating frequency is related to shaft rotation. This signature is present in each CSC map, the most significant modulation is visible in pictures related to the left upper bearing. The second source of modulation is related to the faulty bearing. The fault frequency is equal to 141 Hz. The clearest picture is related to the lower bearing in the vertical direction. The fundamental frequency and two harmonics are seen. As the fault signature is rather weak, it is not visible on other maps (from other sensors).

The situation completely changes after the replacement of the bearings. Additionally, the problem of cyclic shocks was also solved. So, in Figure 11, there are almost empty maps and no modulation (just fundamental rotating frequency). This is evidence for the no-damage case.

The CSC maps are very advanced and frequently used by scientists, however, in engineering practice, the envelope spectrum is preferred. As was shown by Randall et al. [52], the Envelope Spectrum is equivalent to an integrated CSC map.

**Figure 12:**
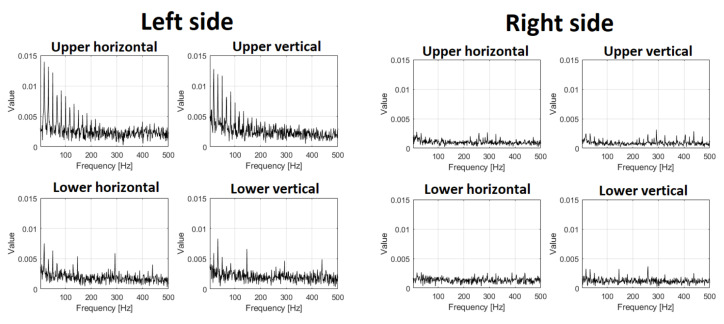
Envelope spectrum before repair.

**Figure 13:**
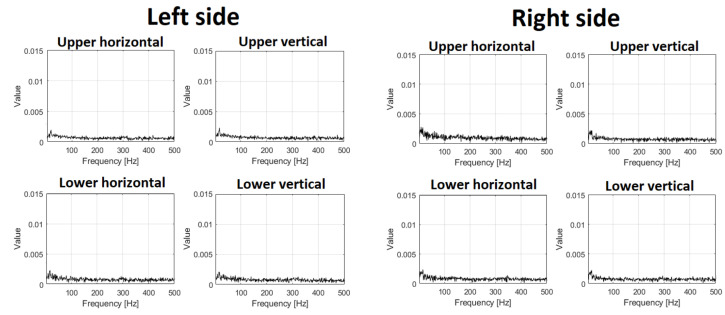
Envelope spectrum after repair.

In Figure 12 and Figure 13, envelope spectra for each sensor (the same layout) are presented. Envelope spectra for the upper left bearing contain a family of harmonics corresponding to rotating frequency. As we present all spectra in the same scale (Y axis), the right side of the screen has almost nothing in the envelope spectrum (shocks are transmitted but with much lower amplitudes). The spectrum for an envelope from the lower left bearing contains some components, so detection of the damage is possible, but they are not so clear for the CSC map.

The results of statistical data analysis by different methods in the frequency domain are represented in Table 1. The quantitative parameter for time series RMS value is taken; for spectrograms, the median energy; for CSC, the ratio of the amplitude of modulating component at fundamental frequency $\left(f_{d}\right)$ to the amplitude at the fundamental frequency of the main operating cycle $\left(f_{c}\right)$ at the resonant center frequency; and for envelope spectrum, the average amplitudes of the 2nd and 3rd harmonics of the main frequency. The time series showed changes in RMS by −68%; median energy of Spectrogram by 89%; amplitudes ratio of CSC by −85%; and average amplitude of harmonics in envelope spectrum by −80%. Hence, the most sensitive method in the frequency domain is based on spectrogram and CSC analysis. These parameters can be used as the “health indicators” in condition monitoring systems of vibrating machines.

**Figure 14:**
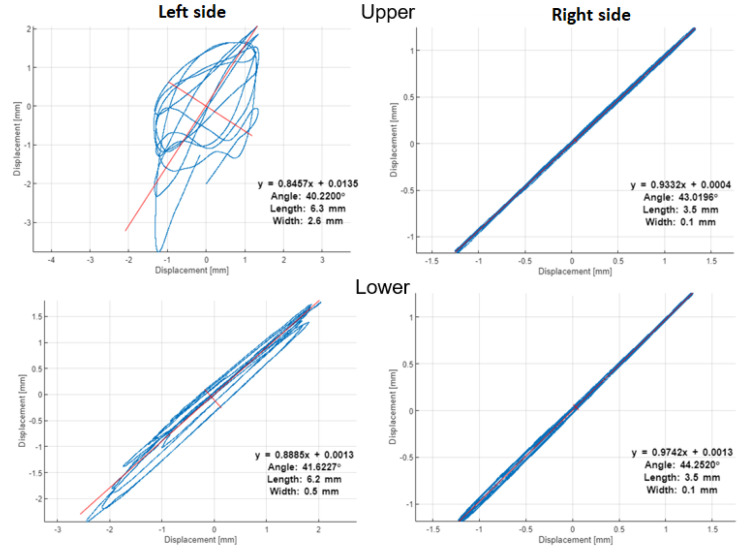
Orbits before repair.

**Figure 15:**
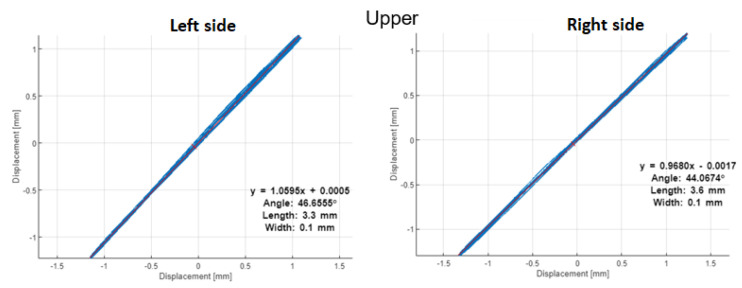

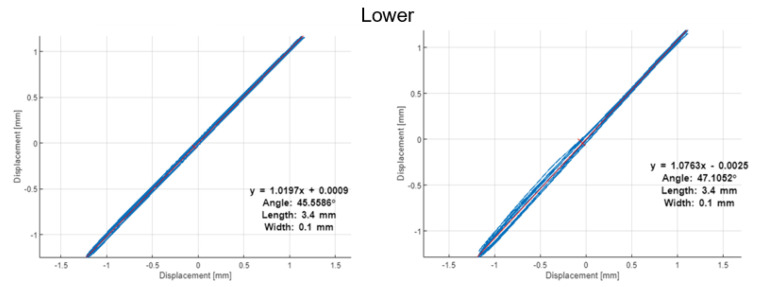
Orbits after repair.

**Figure 16:**
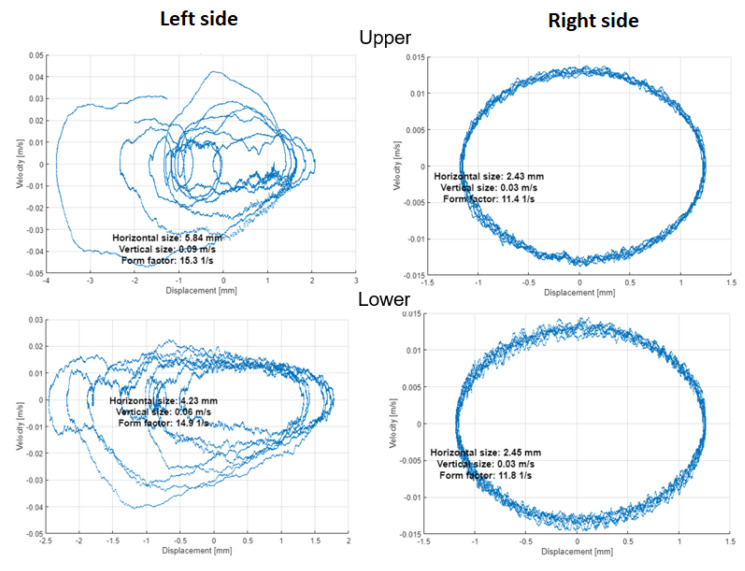
Phase space plots (PSP) before repair.

**Figure 17:**
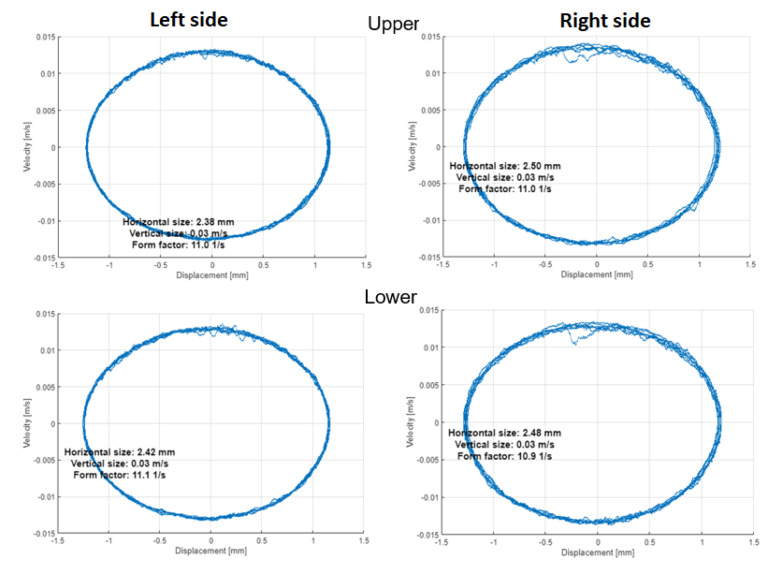
Phase space plots (PSP) after repair.

The orbits in Figure 14 and Figure 15 with PSP in Figure 16 and Figure 17 are built by the vibration signals recorded before bearing replacement and right after repair, respectively. The difference is visible between the trajectories of shaft motion affected by the damaged shield on the upper left support and the bearing damage on the lower left bearing. Since all corresponding graphs have the same scales on both axes, we can conclude that external excitation from the vibrator’s impacts on the shield affects both the orbit and PSP of the upper shaft, while the bearing defect has less effect in the orbit but significantly changes the PSP. The numerical values of orbits and PSP geometry parameters are summarized in Table 2 for the damaged bearing (left side of the lower shaft). The form factor of PSP and angle of orbit inclination are both less sensitive to either the external excitation or the internal bearing defect, because these parameters of dynamics are mainly determined by the screen design (total vibrating mass, supporting springs stiffness, and vibrators’ positions). The size parameters of orbits and PSP were significantly changed due to bearing defect.

After shaft repair and damaged bearing replacement, the orbits and PSP graphs on both sides and both shafts of the screen have no differences in forms and amplitudes of trajectories. Hence, such methods as orbits and PSP can be assumed and easily realized as reliable health indicators in the condition monitoring systems of vibrating machines (not only in sieving screens). It is worth noting that the orbits method needs two orthogonal signals, but PSP needs only one sensor on each bearing.

## 6. Validation

**Figure 18:**
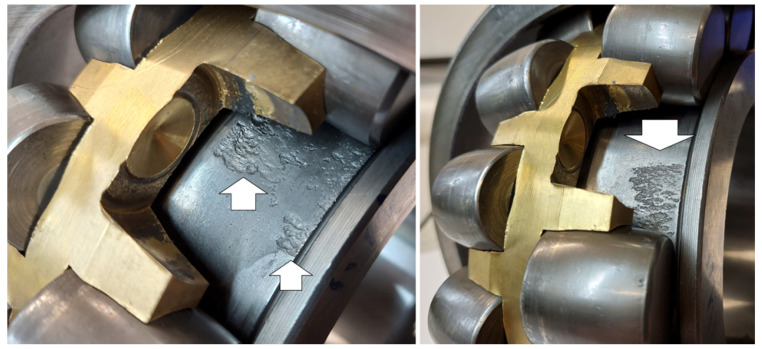
Picture of damaged inner race.

After bearing replacement, we were able to visually investigate the real condition of the bearing elements. According to our findings, the problem is related to the inner race. In Figure 18, pictures of the inner race surface are shown. It is clearly seen that a lot of pits are preset. Definitely, the surface of the inner race is worn. This picture confirms our findings.

The condition of the raceway is also confirmed by the effect observed in the signals: if the damage was of a more local nature (e.g., a transverse fracture), the rolling elements would interact with the failure ones at a time, and we would see distinct short spikes in the time domain in the signals. However, in the case of distributed damage to the surface, several rolling elements are in contact with the damaged surface at any moment in time, so the effect is much more “smoothed” in time than we observed in the signals. Despite this, it did not prevent the identification of the cycle of interaction, and thus the indication of the damaged element.

**Figure 19:**
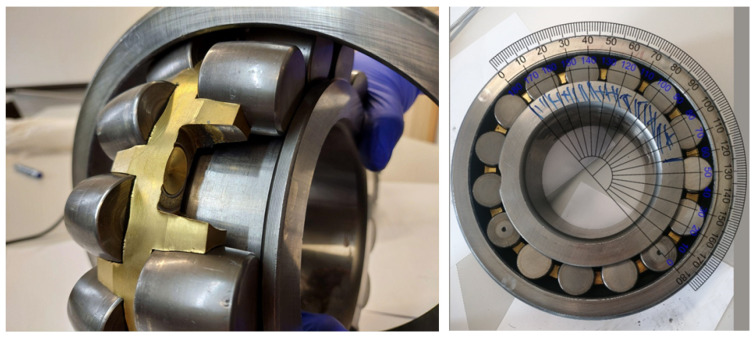
Picture of the not-damaged surface of the inner race and pitting angular size over the ring perimeter.

Figure 19 (left) shows the part of the ring in the healthy condition, which allows comparing its condition with the previous photos (see Figure 18) where pitting occurred over the entire width of the raceway (under both rows of rolling elements). The angular size of pitting is approximately 120°.

The second bearing (the same shaft on the other side), according to the results of the analysis of the signals measured on it, showed no defects, although a thorough inspection of the inner race was complicated due to a different (much more built-in) type of rolling element separator. In the case of the damaged bearing, the situation was much more comfortable, because the type of separator made it possible to remove the barrel with fingers, using the slot provided on the side surface of the raceway. Therefore, no interference with the use of any tools was necessary, and the structure and surface of any of the elements were not affected.

## 7. Discussion

The online calculator available on the SKF website confirms the values of the observed frequencies (see Table 3). The detected defect frequency of about 141 Hz is related to the rolling of the barrels on the inner race, which indicates local damage to the inner race. The replaced (lower) bearing on the day of the second measurement showed noise with energy 39% higher than the upper shaft bearing, which confirms the presence of the temperature difference. Visual inspection confirmed the presence of significant damage to the inner race of the bearing. The damage is in the form of degeneration of the raceway surface (so-called pitting) in the angular range of approximately 1/3 of the entire circumference (almost exactly 120°).

The measurement of the temperature during scheduled inspections can detect the defect in bearings only at late stages of degradation, since it can be related to lubrication oil leaks or jamming.

The measurement of radial clearances in bearings is a time-consuming procedure. The effect of radial clearances is observable at the main frequency of vibrators’ rotation and higher harmonics. Their diagnostics by the vibration signals require further research based on data accumulated during a long period of machine operation.

Future research is aimed at the development of a permanent vibration monitoring system on this vibrating screen. As an additional feature, temperature measurement with contact sensors is recommended, as well as the monitoring of the power (current) of electric motors. The continuous accumulation of vibration data allows for finding trends in health indicators’ progression and adopting diagnostic procedures to detect damages and estimate their sizes more accurately.

## 8. Conclusions

In this paper, an industrial case study related to an inertial vibrator’s bearing fault detection is presented. Thanks to close collaboration with an industrial partner, our research group was lucky to conduct two experiments. We performed vibration measurements in the days before and after the bearing replacement. The reason for maintenance action was related to a slightly higher temperature of the bearings.

A convincing industrial example of the benefits of condition monitoring is not a frequent case in the research literature. Mostly, diagnostic procedures are validated by data from test rigs and seeded defects. Moreover, in very rare cases, authors can present pictures of real damage in bearings.

The data were collected and a comparative analysis was performed by different methods (spectrogram, CSC, envelope spectrum, orbits, and PSP) to highlight the applicability between faulty and healthy machine states.

We were lucky to test advanced signal processing techniques in conditions when extra disturbances (shocks provided by an unbalanced shaft on the damaged shield) are present in measured data before bearing replacement. This phenomenon had a significant impact on signal properties, and consequently proved the reliability of the diagnosis.

Due to advanced methods of data analysis (time–frequency representation, cyclostationary analysis, orbits, and PSP), we were able to identify both high-amplitude cyclic disturbance related to shocks caused by the unbalanced shaft as well as weak cyclic impulses related to the faulty bearing.

After repair, the amplitudes of vibration from the damaged bearing side were reduced by about five times, while sound pressure was only two times lower. This is because the sieving screen itself produces high-level background noise. The quantitative parameters of vibrations showed significant changes: time series RMS (−68%), median energy of spectrograms (89%), frequencies ratio of cyclic spectral coherence (−85%), and average amplitude of harmonics in envelope spectrum (−80%). The orbits demonstrated changes in inclination angle (16%) and sizes (−48, … −96%), as well as PSP sizes (−28, … −67%). the most sensitive method in the frequency domain is based on spectrogram and CSC analysis. These parameters can be used as the "health indicators" in the condition monitoring systems of vibrating machines.

Although amplitude of vibration in time domain showed significant change, the standard spectrum analysis did not allow to localize the defect due to periodic and impulsive noise in the machine. In distinction from previous studies, the quantitative parameters of vibration are calculated, and the corresponding size of the bearing defect is determined. Exceptional satisfaction is related to the visual inspection of the damaged bearing after replacement. Indeed, we identified faulty bearings correctly. Almost 30% of the inner race surface was worn.

The study of this case of bearing failure in the vibrating screen showed that, even under severe periodic disturbances from the vibrators and stochastic impacts from the pieces of sieved material having non-Gaussian distribution (impulsive noise), the concentrated spots of pitting and other local defects in the case of their appearance can be reliably detected.

The main message to the reader is related to the industrial confirmation of the applied diagnostic methods’ efficiency. It is worth investing in condition monitoring systems and advanced signal processing techniques developed by researchers.

The next steps will be in the direction of other defects’ identification and their sizes’ association with quantitative parameters of signals for damage detection at early stages.

## Figures and Tables

**Table 1:** Changes in parameters for different methods on the left side of the lower shaft (damaged bearing).

Methods and Parameters	before Repair	after Repair	Relative Change
**Time Series**			
RMS Value, g	11.71	3.77	−68%
**Time–Frequency Spectrogram**			
Median energy, dB	−27.8	−52.5	89%
**Cyclic Spectral Coherence**			
Amplitudes ratio ($f_{d}/f_{c}$)	1.08	0.16	−85%
**Envelope spectrum**			
Avg. amplitude, $g^{2}$	0.005	0.001	−80%

**Table 2:** Geometric parameters of orbit and PSP on the left side of the lower shaft (damaged bearing).

Geometry Parameters	before Repair	after Repair	Relative Change
**Orbit**			
Angle, grad	40.22	46.65	16%
Length, mm	6.3	3.3	−48%
Width, mm	2.6	0.1	−96%
**PSP**			
Horizontal size, mm	5.84	2.38	−59%
Vertical size, m/s	0.09	0.03	−67%
Form factor (V/H), 1/s	15.3	11.0	−28%

**Table 3:** Characteristic frequencies for SKF 22324 bearing operating at 1000 RPM.

Designation	Frequency [Hz]
Inner ring	16.667
Outer ring	0
Rolling element set and cage	6.853
Rolling element about its axis	44.29
Point on inner ring	147.208
Point on outer ring	102.792
Rolling element	88.579

## Data Availability

The measurement data presented in this study are not publicly available due to restrictions of privacy.
